# Study on Risk Factors for Severe Hand, Foot and Mouth Disease in China

**DOI:** 10.1371/journal.pone.0087603

**Published:** 2014-01-29

**Authors:** Wei Li, Guangju Teng, Hongfei Tong, Yanmei Jiao, Tong Zhang, Hui Chen, Hao Wu

**Affiliations:** 1 Center for Infectious Diseases, Beijing You'an Hospital, Capital Medical University, Beijing, China; 2 Department of Non-Infectious Liver Disorders, 302 Military Hospital of China, Beijing, China; 3 Department of General Surgery, Second Affiliated Hospital of Wenzhou Medical College, Wenzhou, Zhejiang, China; 4 School of Biomedical Engineering, Capital Medical University, Beijing, China; University of Illinois at Chicago, United States of America

## Abstract

**Background:**

Epidemics of HFMD are elevated every year globally, especially in mainland China. The disease now presents as an increasing threat to public health worldwide.

**Methods:**

Five hundred and seventy-one EV71-infected HFMD patients in Beijing You'an Hospital were grouped by disease severity: Mild (no severe complication) (n = 221), and Severe group (complicated with brainstem encephalitis (BE), and/or pulmonary edema (PE) (n = 350)). Clinical and laboratory findings and levels of 7 serum cytokines were analyzed.

**Results:**

Univariate analysis showed that (RR)>26/min (p<0.001), age<4 yo (p = 0.001), GLU>8.3 mmol/L (p = 0.008), CL<98 mmol/L (p = 0.026), and WBC>1.2×10^9^/L (p = 0.040) were associated with severe cases. Results of multivariate analysis indicated five independent risk factors (RR>26/min (p<0.001), Age<4 yo (p<0.001), GLU>8.3 mmol/L (p = 0.011), LYM>40% (p = 0.010), and ALT>40 U/L (p = 0.045)). In addition to single-factor analysis, we further analyzed the use of different combinations of risk factors. “GLU>8.3 and CL<98 and RR>26” (confidence ration (CR) = 100%) is the top indicator, followed by “ALT>40 and LYM>40% and RR>26 and Age<4 yo” (CR = 92.9%).

Serum levels of IL-2, IL-4, IL-10, IFNγ, GM-CSF, and TNFα were higher in severe cases than in mild cases. A new evaluation scoring system by scoring each risk factor 1 and independent risk factor 2 was developed for early identification of severe HFMD cases.

**Conclusions:**

Five independent risk factors, along with indicative combinations of risk factors, for severe cases were identified, and a scoring system was created to facilitate the use of indicators for early medical intervention.

## Introduction

Hand, foot and mouth disease (HFMD) is a common disorder in young children. It is considered as a potentially life-threatening disease[1], [2]. The dominant pathogens for this infectious disease include Coxsachie virus 16 (CA16) and Enterovirus 71 (EV71). Cases with EV71-positive carry a higher mortality rate if compared with those with CA16. Most of major HFMD outbreaks in recent years were caused by EV71, which is a member of the *Enterovirus* genus in the *Picornavirus* family. Although HFMD could be seen globally, Mainland China is one of the main regions where EV71 outbreaks could be seen, in almost every year recently[2], [3], [4], [5], [6], [7]. The clinical manifestations of most HFMD cases were mild and limited to fever and vesicular exanthema on patients' palms, soles, and mouth along with discomfortness at certain levels. These mild cases are generally self-limited and not life-threatening. However, the incidence of severe cases is not low, especially in mainland China. Severe cases with potentially fatal complications such as brain stem encephalitis (BE) and/or pulmonary edema (PE) may lead to serious sequelae, even death[2], [8].

Accumulating evidence from global reports on HFMD epidemics supports the fact that the ratio of severe cases is elevating gradually, along with mortality rate[9]. Finding good early clinical and/or serological indicators to identify potential severe cases would be an effective way to provide supports for early medical intervention on particular cases and reduce mortality. However, until now, no reliable markers have been identified. Previous reports indicate that hyperglycemia and leukocytosis have been found to be elevated in severe HFMD patients[10], [11], [12]. It also has been reported that cytokines may play critical roles in the pathogenesis of EV71 infection[13], [14], [15], [16], [17]. Studies on cytokine levels showed that levels of many cytokines, such as interferon gamma (IFNγ), interleukin-1β (IL-1β), IL-1Rα, IL-6, IL-10, IL-13, granulocyte colony-stimulating factor (G-CSF) and tumor necrosis factor alpha (TNFα) in serum and cerebral spinal fluid (CSF) were elevated in severe cases[14], [16], [18], [19]. But these reports are often inconclusive, and identified potential markers are not specific enough to support early clinical intervention. It is still an open question on what could be reliable markers to indicate early treatment.

The objective of this study was to analyze the clinical and laboratory data of a group of pediatric HFMD patients admitted to Beijing You'an Hospital, Capital Medical University, and evaluate the correlations between early clinical-laboratory findings and disease severity. This study aims to identify early indicators of disease severity so that prophylactic measures can be taken to reduce mortality. In addition to evaluate individual markers, we tried to use different combinations of markers as indicators of severity.

## Materials and Methods

### Case definition

The case definition is described elsewhere[18]. Briefly, EV71 infection was defined as the isolation of the virus from at least 1 site (throat swab, blood, stool, cerebrospinal fluid (CSF), or other) with a negative bacterial culture. BE was defined as a disease characterized by myoclonus, ataxia, nystagmus, oculomotor palsies, and bulbar palsy in various combinations, with or without neuroimaging. PE was defined as respiratory distress with tachycardia, tachypnea, rales, with or without frothy sputum, and a positive chest radiograph that showed pulmonary infiltrates without cardiomegaly.

### Study population

Information of the study population consisted of 571 children who met the case definition described above was collected retrospectively. The patients were consecutively admitted to Beijing You'an Hospital, Capital Medical University (Beijing, PR.China) between Mar and Oct, 2012. All these cases were tested for serum cytokine levels.

Patients were divided into two categories based on severity: (1) Mild: patients with HFMD without BE or PE; (2) Severe: HFMD patients complicated by BE or/and PE.

### Data source

All of the parameters included in the investigation were collected by reviewing the patient medical records that were preserved in the medical record library and the medical computerized database at Beijing You'an Hospital, Capital Medical University. The patient records were retrospectively examined for primary set of data, which included demographic characteristics (age and sex), clinical parameters (signs and symptoms), laboratory values (hematologic, biochemical and microbiological findings), radiologic data, outcome at discharge (recovered or died), admission and discharge dates, and length of hospital stay. Clinical findings and laboratory results analyzed in this study were from the examination done right upon admission of all involved patients.

You'an Hospital Ethics Committee has approved this study. It covered the retrospective analysis of the 571 records. Parents or caretakers of all 571 participants have given a written informed consent on behalf of the children participants for their information to be stored and used for research. Human experimentation guidelines of PR.China were followed in the conduct of this clinical research.

### Cytokine level determination

The blood samples for cytokine determination were collected immediately upon patient admission. The plasma was harvested within 30 min at 37°C of venipuncture from EDTA-anticoagulated blood samples and stored at −70°C until analyzed. The Bio-Plex Human 8-plex kit (Bio-Rad, USA) was used to detect IL-2, IL-4, IL-6, IL-10, IFNγ, GM-CSF and TNFα levels on Luminex200 xMAP analyzer system (Luminex, USA), according to the manufacturer's instructions.

### Statistical analysis

Average of data was presented as 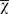
±SD. Categorical data were tested by use of χ^2^ or Fisher's exact test. Continuous data were analyzed by using Spearman's rho correlation analysis. Mann-Whitney U test was used for data that did not have a normal distribution. Correlation among cytokine levels was analyzed by hierarchical cluster analysis. All analyses were performed by SPSS software (version 10.0, SPSS). P value <0.05 was considered to be statistically significant.

## Results

### Patient characteristics

The demographic and clinical data of 571 HFMD patients are collected and summarized in **Table 1**. The average time from the appearance of the first symptom (fever or rash) to hospital admission was 1.59±2.10 days. Patients in both groups were recovered and discharged, except 2 children in severe cases who died.

**Table 1:** Patient characteristics.

	Total (n = 571)	Mild (n = 221)	Severe (n = 350)
Age (year)	2.58±1.80	3.64±1.70	1.87±1.98
Gender (Male/Female)	351/220	151/70	200/150
Weight (kg)	14.06±6.60	18.11±7.89	12.56±8.86
RR (/min)	28.14±5.63	26.10±6.13	32.01±6.76
Temperature (°C)	37.67±0.92	36.54±0.77	38.23±0.78
Pulse (/min)	123.12±16.83	117.34±16.56	129.61±18.99
Systolic pressure (mmHg)	101.45±11.35	95.33±21.33	107.54±23.54
Diastolic pressure (mmHg)	69.99±9.05	62.66±13.51	77.76±31.45
GLU (mmol/L)	6.30±2.50	5.52±3.12	7.83±2.92
WBC (10^9^/L)	11.18±4.43	9.33±3.12	13.83±4.24
Neutrophils (%)	56.45±18.11	51.09±20.65	60.09±22.94
Lymphocytes (%)	35.94±16.52	33.21±21.21	38.46±17.92
HB (g/dL)	119.08±12.34	118.92±22.01	119.66±31.28
PLT (10^9^/L)	313.23±91.44	310.11±98.10	312.92±101.31
ALT (IU/L)	21.86±35.21	20.21±30.22	23.54±29.35
AST (IU/L)	37.91±21.06	35.87±20.92	37.84±32.12
CK (IU/L)	144.11±193.71	152.42±200.12	142.98±188.72
CK-MB (IU/L)	16.51±26.20	20.48±21.32	15.89±34.02
CRP (mg/L)	3.61±4.14	3.24±5.22	3.77±3.89
LDH (U/L)	260.17±74.62	258.11±85.32	268.39±74.66
K (mmol/L)	4.29±1.02	4.35±1.08	4.22±1.31
Na (mmol/L)	136.17±3.76	138.56±3.98	135.09±4.22
CL (mmol/L)	101.67±41.80	104.27±40.96	95.38±39.69

RR: respiratory rate, GLU: blood glucose, LYM: percentage of lymphocytes, ALT: alanine aminotransferase, CL: blood chlorine, WBC: white blood cell counts, CK: creatine kinase, CK-MB: creatine kinase-MB, CRP: C-reactive protein, LDH: Lactate dehydrogenase.

### Risk factors for severe HFMD cases

**Figure 1:**
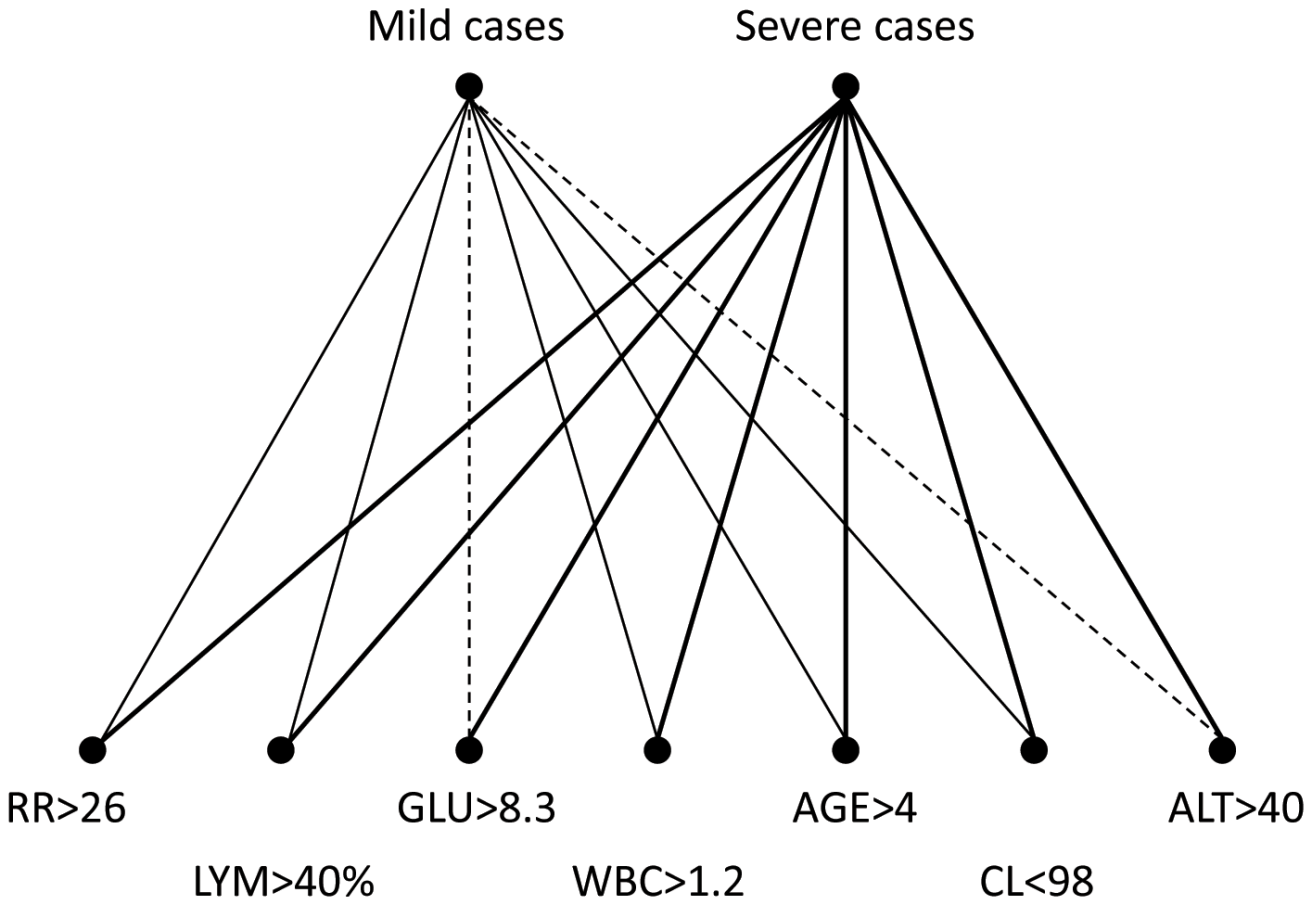
Directed correlation web of risk factors and disease severity. The percentages of coexistence of two factors among all selected cases were shown in the form of directed correlation web. The line thickness indicates the rates of coexistence. Bold line: Rate of coexistence of two linked factors among all cases is >50%; Thin line: Rate of coexistence of two linked factors among all cases is 30%–50%; Dash line: Rate of coexistence of two linked factors among all cases is <30%. RR: respiratory rate (/min), GLU: blood glucose (mmol/L), LYM: percentage of lymphocytes (%), ALT: alanine aminotransferase (U/L), CL: blood chlorine (mmol/L), WBC: white blood cell counts (10^9^/L).

After screening clinical and laboratory parameters by using univariate analysis, we identified five risk factors for severe HFMD cases: (RR)>26/min (p<0.001), age<4 yo (p = 0.001), GLU>8.3 mmol/L (p = 0.008), CL<98 mmol/L (p = 0.026), and WBC>1.2×10^9^/L (p = 0.040). Results of multivariate analysis indicated five independent risk factors (RR>26/min (p<0.001), Age<4 yo (p<0.001), GLU>8.3 mmol/L (p = 0.011), LYM>40% (p = 0.010), and ALT>40 U/L (p = 0.045)) (**Table 2**). To further visualize the correlation between disease severity and seven risk factors, we created a directed correlation web based on the rates of co-existence of mild or severe cases and different risk factors (**Figure 1**). The strongest links were all seen with severe cases, which were above 63%. The best link was seen between severe cases and Glu>8.3 (82.05%), followed by severe cases and ALT>40 (78.26%). The links with mild cases were all <37%, among which the best was with age<4 yo (36.45%), and the worst two were with ALT>40 (21.74%) and Glu>8.3 (17.95%).

**Table 2:** Risk factors for severe HFMD cases.

	P value	
	Univariate analysis	Multivariate analysis
RR>26/min	<0.001	<0.001
Age<4 yo	0.001	<0.001
GLU>8.3 mmol/L	0.008	0.011
LYM>40%	0.075	0.010
ALT>40 U/L	0.099	0.045
CL<98 mmol/L	0.026	
WBC>1.2×10^9^/L	0.040	
Fever>38.5°C	0.055	

RR: respiratory rate, GLU: blood glucose, LYM: percentage of lymphocytes, ALT: alanine aminotransferase, CL: blood chlorine, WBC: white blood cell counts.

### Patterns of risk factors in combinations

Using single parameter to predict disease severity is not a reliable and accurate way to assist diagnosis and initiate early intervention. Therefore, we analyzed various combinations of different important parameters we found above. We tested the positive rates of different combinations of the seven markers and calculated Popularity, Confidence ratio (CR) and Support (**Table 3**). Popularity is the chance of seeing the combination among all cases. CR is the percentage of severe cases among cases with the combination. Support equals Popularity x CR, which indicates the ratio of severe cases with the combination out of all cases.

**Table 3:** Combinations of risk factors.

Combinations	Case# (n)	Popularity (%)	CR (%)	Support (%)
GLU>8.3 and CL<98 and RR>26	12	2.1	100.0	2.1
ALT>40 and LYM>40% and RR>26 and Age<4 yo	14	2.5	92.9	2.3
GLU>8.3 and CL<98	16	2.8	87.5	2.5
GLU>8.3 and WBC>1.2 and RR>26	16	2.8	87.5	2.5
GLU>8.3 and RR>26	31	5.5	87.1	4.8
GLU>8.3 and LYM>40% and RR>26	23	4.1	87.0	3.5
ALT>40 and LYM>40% and RR>26	15	2.6	86.7	2.3
GLU>8.3 and WBC>1.2 and RR>26 and Age<4 yo	15	2.6	86.7	2.3
GLU>8.3 and CL<98 and Age<4 yo	14	2.5	85.7	2.1
GLU>8.3 and RR>26 and Age<4 yo	28	4.9	85.7	4.2
GLU>8.3 and WBC>1.2 and LYM>40% and RR>26	14	2.5	85.7	2.1
GLU>8.3 and LYM>40% and RR>26 and Age<4 yo	21	3.7	85.7	3.2
GLU>8.3 and WBC>1.2 and LYM>40% and RR>26 and Age<4 yo	13	2.3	84.6	1.9
GLU>8.3 and LYM>40%	31	5.5	83.9	4.6
ALT>40 and RR>26 and Age<4 yo	24	4.2	83.3	3.5
GLU>8.3 and WBC>1.2 and LYM>40%	18	3.2	83.3	2.6
CL<98 and WBC>1.2 and LYM>40% and RR>26	23	4.1	82.6	3.4
ALT>40 and LYM>40% and Age<4 yo	17	3.0	82.4	2.5
GLU>8.3 and WBC>1.2 and LYM>40% and Age<4 yo	17	3.0	82.4	2.5
GLU>8.3 and LYM>40% and Age<4 yo	28	4.9	82.1	4.1
CL<98 and WBC>1.2 and LYM>40% and RR>26 and Age<4 yo	22	3.9	81.8	3.2
GLU>8.3 and WBC>1.2	21	3.7	81.0	3.0
CL<98 and LYM>40% and RR>26 and Age<4 yo	73	12.9	80.8	10.4
CL<98 and WBC>1.2 and LYM>40%	31	5.5	80.6	4.4
ALT>40 and RR>26	25	4.4	80.0	3.5
GLU>8.3 and WBC>1.2 and Age<4 yo	20	3.5	80.0	2.8
GLU>8.3	43	7.6	79.1	6.0
CL<98 and WBC>1.2 and LYM>40% and Age<4 yo	28	4.9	78.6	3.9
ALT>40 and WBC>1.2	18	3.2	77.8	2.5
ALT>40 and LYM>40%	18	3.2	77.8	2.5
LYM>40% and RR>26 and Age<4 yo	191	33.7	77.5	26.1
WBC>1.2 and LYM>40% and RR>26 and Age<4 yo	75	13.2	77.3	10.2
ALT>40 and WBC>1.2 and RR>26	13	2.3	76.9	1.8
ALT>40 and WBC>1.2 and RR>26 and Age<4 yo	13	2.3	76.9	1.8
ALT>40 and WBC>1.2 and Age<4 yo	17	3.0	76.5	2.3
GLU>8.3 and Age<4 yo	38	6.7	76.3	5.1
CL<98 and LYM>40% and RR>26	80	14.1	76.3	10.8
CL<98 and LYM>40% and Age<4 yo	92	16.2	76.1	12.3
ALT>40 and Age<4 yo	33	5.8	75.8	4.4
WBC>1.2 and LYM>40% and RR>26	82	14.5	75.6	10.9
CL<98 and RR>26 and Age<4 yo	111	19.6	73.9	14.5
CL<98 and WBC>1.2 and RR>26	38	6.7	73.7	4.9
WBC>1.2 and RR>26 and Age<4 yo	127	22.4	73.2	16.4
LYM>40% and RR>26	215	37.9	73.0	27.7
CL<98 and WBC>1.2 and RR>26 and Age<4 yo	37	6.5	73.0	4.8
WBC>1.2 and RR>26	134	23.6	72.4	17.1
ALT>40	36	6.3	72.2	4.6
CL<98 and WBC>1.2	54	9.5	72.2	6.9
WBC>1.2 and LYM>40% and Age<4 yo	104	18.3	72.1	13.2
CL<98 and LYM>40%	111	19.6	72.1	14.1
CL<98 and RR>26	119	21.0	71.4	15.0
RR>26 and Age<4 yo	336	59.3	71.4	42.3
LYM>40% and Age<4 yo	265	46.7	71.3	33.3
WBC>1.2 and LYM>40%	118	20.8	70.3	14.6
CL<98 and WBC>1.2 and Age<4 yo	50	8.8	70.0	6.2

RR: respiratory rate (/min), GLU: blood glucose (mmol/L), LYM: percentage of lymphocytes (%), ALT: alanine aminotransferase (U/L), CL: blood chlorine (mmol/L), WBC: white blood cell counts (10^9^/L), CR: Confidence ratio

The most reliable combination is “GLU>8.3 and CL<98 and RR>26”, which offered 100% CR, which means all patients with this combination belonged to severe cases. The second most reliable combination is “ALT>40 and LYM>40% and RR>26 and Age<4 yo”, whose CR was 92.9%. Among the top 10 combinations, there are two very simple ones, “GLU>8.3 and CL<98” and “GLU>8.3 and RR>26”, whose CR were 87.5% and 87.1%, respectively. The combination “RR>26 and Age<4 yo” gave the best Popularity (59.3%), which came with the highest Support (42.3%) and a CR of 71.4%.

### Correlations between risk factors

**Figure 2:**
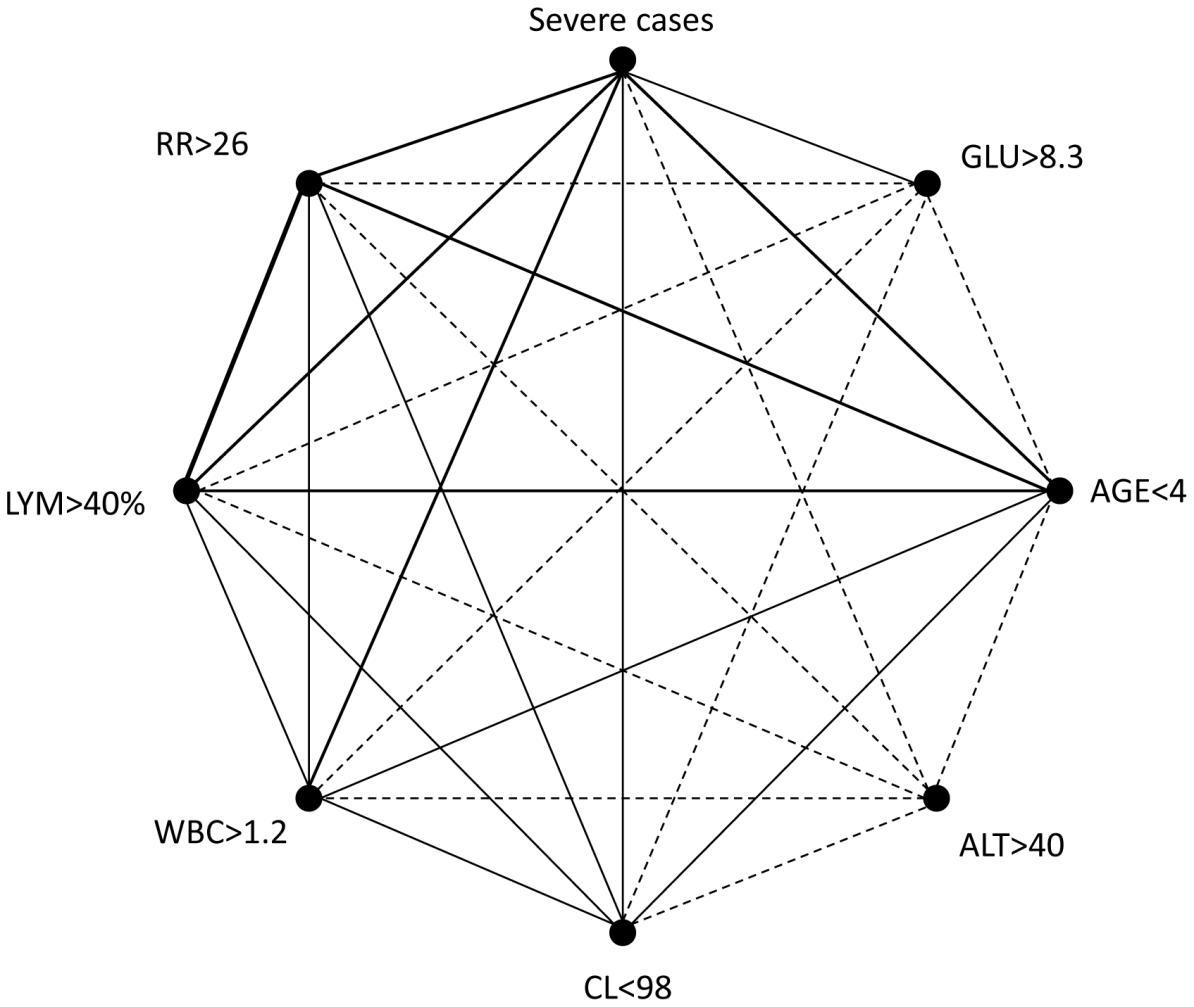
Correlation web on correlations among risk factors and severe cases. The percentages of coexistence of two factors among all selected cases were shown in the form of correlation web. The line thickness indicates the rates of coexistence. Bold line: Rate of coexistence of two linked factors among all cases is >40%; Thin line: Rate of coexistence of two linked factors among all cases is 15%–40%; Dash line: Rate of coexistence of two linked factors among all cases is <15%. RR: respiratory rate (/min), GLU: blood glucose (mmol/L), LYM: percentage of lymphocytes (%), ALT: alanine aminotransferase (U/L), CL: blood chlorine (mmol/L), WBC: white blood cell counts (10^9^/L).

To further analyze the links between risk factors and severe cases, and correlations among risk factors, we created a correlation web (**Figure 2**) based on the rates of co-existence of mild or severe cases and different risk factors. The top five links were seen with RR>26 and Age<4 (70.9%), RR>26 and severe cases (69.03%), LYM>40% and severe cases (65.12%), Age<4 and severe cases (63.55%), and RR>26 and LYM>40% (61.06%). The worst five were all seen with ALT>40 (with severe cases (8.57%), RR>26 (7.52%), Age<4 (7.36%), LYM>40% (5.58%) and CL<98 (4.59%)).

### Plasma cytokine levels of HFMD patients

Cytokines are among the key mediators of immune responses to invasion of virus and other pathogens. Therefore, the levels of IL-2, IL-4, IL-6, IL-10, IFNγ, GM-CSF, and TNFα in all enrolled subjects were tested upon admission, and results are presented in **Table 4**. Except for IL-6, levels of all other tested cytokines were significantly higher in severe cases than in mild cases.

**Table 4:** Correlations between cytokine levels and disease severity.

	Mild (n = 221)			Severe (n = 350)			P value
	Median	Quartile range		Median	Quartile range		
IL2	5.33	3.25	9.13	9.40	4.09	16.23	0.006
IL4	0.68	0.49	1.02	0.90	0.66	1.26	0.003
IL6	11.90	6.54	22.97	18.02	6.89	43.61	0.104
IL10	5.90	3.98	11.65	8.11	5.04	24.33	0.041
IFNγ	70.92	43.96	182.86	124.62	86.53	198.85	0.004
GMCSF	14.06	10.93	28.15	18.95	12.26	30.77	0.029
TNFα	17.00	13.07	39.23	30.04	15.27	88.34	0.008

### Scoring system for severe HFMD cases

Based on our analysis, we tried to create a practical evaluation system for severe HFMD cases, since most combinations with high CR came with a very low popularity, indicating that using only CR as an indicator for severe cases was not practicable (**Table 3**). According to results of statistical analysis, each risk factor in Table 2 is given a score 1, and each independent risk factor scores 2. In our study, the highest calculated score for a combination with a CR>70% was found to be 9 and the lowest was 2. The calculated scores in our system were positively associated with CR (p<0.001), support (p = 0.001) and popularity significantly (p = 0.001), which indicated that the higher the score, the worse the case should be.

## Discussion

HFMD is a potential life-threatening illness and finding early markers to identify potential dangerous cases could reduce mortality rate significantly. In our study, respiratory rate (RR)>26/min, blood glucose (GLU)>8.3 mmol/L, age<4 yo, percentage of lymphocytes (LYM)>40%, blood chlorine (CL)<98 mmol/L, and white blood cell counts (WBC)>1.2×10^9^/L were found to be associated with severe cases. And results of multivariate analysis indicated five independent risk factors (RR>26/min, Age<4 yo, GLU>8.3 mmol/L, LYM>40%, and ALT>40 U/L) (**Table 2**). Previous publications have pointed out that GLU, age, sustained fever and high WBC were significant risk factors for severe HFMD cases[12], [20]. Our data indicated that RR, high lymphocyte ratio, high ALT and low CL may also linked to severe diseases.

In addition to single-factor analysis, we further analyzed the use of different combinations of risk factors. Based on 3 parameters (popularity, confidence ratio and support), combinations of risk factors were evaluated. Combinations with the best confidence ratio should be the best indicator for severe cases by definition. Therefore, “GLU>8.3 and CL<98 and RR>26” (CR = 100%) is our top indicator, followed by “ALT>40 and LYM>40% and RR>26 and Age<4 yo” (CR = 92.9%). All combinations with a CR> = 80% came with a case number<32 and a support<4.9%, except “CL<98 and LYM>40% and RR>26 and Age<4 yo”, whose case number was 73 and support was 10.4%. On the contrary, combinations with a lower CR (70.0% to 79.9%) had a much higher case number and support in average, if compared with those with a CR>80.0%. Among combinations with a lower CR, 37.9% (11/29) had a case number>100 (17.5% of all enrolled cases), and 48.3% (14/29) had a support>10%. This implies that these combinations are more commonly seen in clinical practice and should not be ignored. Although combinations with high CR may help identifying severe cases, their popularity is very low (**Table 3**). So a scoring system was developed for clinicians based on our data. The statistical analysis favored our system and implied that it may benefit severe HFMD patients. However, further evaluation on this system is required to ultimately validate its clinical use.

Prior studies have demonstrated the correlation between certain cytokines and HFMD. The study on ratio of cytokine levels in CSF over in plasma had suggested the possible role of IL-8 in response to EV71 infection complicated with PE[21]. Serum IL-1, IL-6 and IFNγ were higher in HFMD patients with PE than in patients without[16]. IL-1β, IL-1Rα, and G-CSF are recently reported by Griffiths et al as prognostic markers for EV71-infected hospitalized Malaysian children[19], [22]. In our study, except for IL6, levels of IL2, IL4, IL10, IFNγ and TNFα were all significantly higher in severe cases than in mild ones (**Table 3**).

In conclusion, five independent risk factors, along with indicative combinations of risk factors, for severe cases were identified, and a scoring system was created to facilitate the use of indicators for early medical intervention.
